# Rational Design of Multi‐Color‐Emissive Carbon Dots in a Single Reaction System by Hydrothermal

**DOI:** 10.1002/advs.202001453

**Published:** 2020-11-23

**Authors:** Boyang Wang, Jingkun Yu, Laizhi Sui, Shoujun Zhu, Zhiyong Tang, Bai Yang, Siyu Lu

**Affiliations:** ^1^ Green Catalysis Center and College of Chemistry Zhengzhou University Zhengzhou 450000 China; ^2^ State Key Lab of Molecular Reaction Dynamics Dalian Institute of Chemical Physics Chinese Academy of Sciences Dalian 116023 China; ^3^ State Key Lab of Supramolecular Structure and Materials College of Chemistry Jilin University Changchun 130012 China; ^4^ Henan Institute of Advanced Technology Zhengzhou University Zhengzhou 450000 China; ^5^ CAS Key Laboratory of Nanosystem and Hierarchical Fabrication CAS Center for Excellence in Nanoscience National Center for Nanoscience and Technology Beijing 100190 China

**Keywords:** carbon dots, fluorescence mechanism, multi‐color‐emissive, phosphors, two‐photon fluorescence

## Abstract

As an emerging building unit, carbon dots (CDs) have been igniting the revolutionaries in the fields of optoelectronics, biomedicine, and bioimaging. However, the difficulty of synthesizing CDs in aqueous solution with full‐spectrum emission severely hinders further investigation of their emission mechanism and their extensive applications in white light emitting diodes (LEDs). Here, the full‐color‐emission CDs with a unique structure consisting of *sp* ^3^‐hybridized carbon cores with small domains of partially *sp* ^2^‐hybridized carbon atoms are reported. First‐principle calculations are initially used to predict that the transformation from *sp* ^3^ to *sp* ^2^ hybridization redshifts the emission of CDs. Guided by the theoretical predictions, a simple, convenient, and controllable route to hydrothermally prepare CDs in a single reaction system is developed. The prepared CDs have full‐spectrum emission with an unprecedented two‐photon emission across the whole visible color range. These full‐color‐emission CDs can be further nurtured by slight modifications of the reaction conditions (e.g., temperature, pH) to generate the emission color from blue to red. Finally a flexible LEDs with full‐color emission by using epoxy CDs films is developed, indicating that the strategy affords an industry translational potential over traditional fluorophores.

## Graphical abstract

A novel multi‐color‐emissive carbon dots (CDs) with two‐photon fluorescence are developed in aqueous solution for the first time by slight modifications of the reaction conditions. Theoretical calculations combined with experiments show that the photoluminescence mechanism of the multi‐color‐emissive CDs is the size change and the conversion of *sp* ^3^ to *sp* ^2^ hybridization.

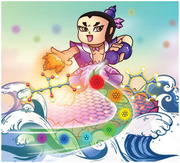

## 1 Introduction

Materials with multi‐color photoluminescence (PL) that are excited by a single wavelength are of particular research interest because of their potential usages in light emitting diodes (LEDs), bioimaging, and optoelectronics.^[^ ^1^, ^2^, ^3^, ^4^ ^]^ Among them, LEDs have attracted particular attentions for the potential applications in multi‐color display, low‐cost back‐lighting in liquid‐crystal displays, and next‐generation lighting sources for our daily life. The typical materials developed in these areas include molecular nanomaterials,^[^ ^5^, ^6^ ^]^ rare‐earth‐based nanoparticles,^[^ ^7^, ^8^ ^]^ semiconductor quantum dots,^[^ ^9^, ^10^ ^]^ and organic fluorescent dyes.^[^ ^11^, ^12^ ^]^ However, low emission quantum yields (QYs), susceptibility to photobleaching, and complicated fabrication have limited the wide adoption of these materials.

Carbon dots (CDs) show unique PL properties and have a few exotic characteristics including high stability, biocompatibility, ease of modification, and low cost, which enable CDs to wide uses in different fields.^[^ ^13^, ^14^, ^15^ ^]^ However, a major difficulty is to prepare long‐wavelength, two‐photon emission, and fully emissive CDs. Currently, several kinds of CDs with full‐spectrum emission have been synthesized and used in various applications.^[^ ^16^, ^17^, ^18^, ^19^, ^20^, ^21^ ^]^ For examples, Lin et al. prepared CDs with blue, green, or red emission by modifying the carbon source (*o*‐, *m*‐, or *p*‐phenylenediamine).^[^ ^16^ ^]^ Xiong et al. reported the one‐pot synthesis of multi‐color CDs with blue to red emission that were separated by column chromatography.^[^ ^17^ ^]^ Sun et al. used controlled graphitization and surface functionalization to obtain a series of multi‐color CDs. Although these studies yield bright PL‐tunable CDs, the synthetic methods are still complicated and require different precursors and solvents.^[^ ^18^ ^]^

To our best knowledge, until now, no experimental work reports full‐color emissive CDs by using only one set of precursors in water. The underlying emissive mechanisms of these CDs are still controversial.^[^ ^19^, ^20^, ^21^ ^]^ Particularly, when the samples have complex compositions, the proposed PL mechanisms are not convincing because of the many influencing factors exited. Therefore, it is necessary to develop a simple and convenient synthetic strategy for the aqueous preparation of full‐color emissive CDs with a clear and common mechanism.

This work describes CDs integrating *sp* ^3^‐hybridized carbon cores with small domains of partially *sp* ^2^‐hybridized carbon atoms. Our theoretical prediction suggests that increasing the number of strongly *sp* ^2^‐hybridized domains in the CDs cores lead to the emission to longer wavelengths in the visible spectrum for such a type of CDs. On this basis, we develop a facile, green, and controllable synthetic approach to prepare full‐spectrum emissive CDs in one system without changing the precursors or solvent. This is the first report on full‐spectrum emissive CDs with simultaneous two‐photon fluorescence by using a hydrothermal method. Moreover, we further develop a universal mechanism to explain the observed full‐spectrum emission, in which the optical properties of the *sp* ^2^/*sp* ^3^ hybridized CDs are mainly determined by the combined effects of particle size and the partially *sp* ^2^ hybridized carbon domains in the *sp* ^3^‐hybridized cores.

## 2 Results and Discussion

**Figure 1:**
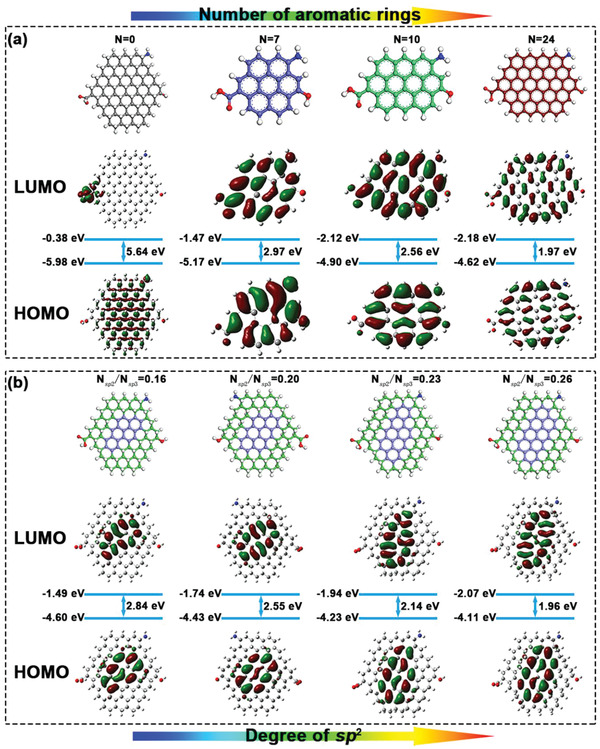
a) HOMO and LUMO states of the established model by increasing the aromatic rings; b) HOMO and LUMO states of the established model by increasing the ratio of *sp* ^2^/*sp* ^3^ hybridized domains.

In this study, we propose the optical responses of the multi‐color‐emissive CDs can be achieved by a simple, accurate, semi‐analytical model of the CDs optical centers.^[^ ^22^, ^23^ ^]^ Our proposed CDs are considered as *sp* ^3^‐hybridized carbon cores with small domains of *sp* ^2^‐hybridized carbon and systematically investigated by density functional theory (DFT) calculations. The size effects of the CDs on the fluorescence emission are assessed by designing several *sp* ^2^‐hybridized benzene ring structures. CDs with similar internal structures, but with increasing numbers of benzene rings, and thus increasing size, are compared. As show in **Figure** **1**a, the increasing size redshift the excitation wavelength gradually. The smallest CDs emit at 417.12 nm, whereas the larger CDs emit at 626.87 nm (Table S1, Supporting Information), indicating that the emission wavelengths can cover the entire visible spectrum. The redshift is attributed to narrowing band‐gap caused by p electron delocalization. In addition to the size effect, the *sp* ^2^/*sp* ^3^ hybridized domain is also closely related to luminescence (Table S2, Supporting Information). Regardless of size, if there is no *sp* ^2^ hybridization, the emission wavelength should not be red‐shifted. For CDs with similar size, increasing the degree of internal *sp* ^2^ hybridization induces the red‐shifts emission, suggesting that the fluorescence emission of the CDs depends on both the size and the *sp* ^2^/*sp* ^3^ hybridized domains, as illustrated in Figure 1b.

**Figure 2:**
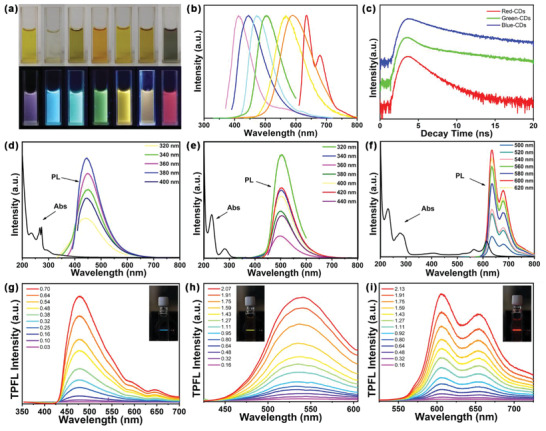
a) Photos of the as‐prepared CDs in daylight (upper) and UV light (bottom), respectively; b) Normalized PL emission spectra of all the above samples under excitation of 365 nm; c) Time‐resolved PL spectra of the four selected samples. Absorption spectra and PL emission spectra of d) B‐CDs, e) G‐CDs, and f) R‐CDs under excitation of different wavelengths of light. Two‐photon spectra with different laser powers of 800 nm femtosecond pulse laser of g) B‐CDs, h) G‐CDs, and i) R‐CDs (the inset photograph is under 800 nm excitation).

Above theoretical predictions were tested by selecting the precursors citric acid (CA), which contains a *sp* ^3^ hybridized structure, and *o*‐phenylenediamine (oPD), which contains a *sp* ^2^ hybridized structure. Multi‐color‐emissive CDs that show tunable PL emissions from deep blue to near‐infrared (NIR) were hydrothermally synthesized by heating CA and oPD in deionized (DI) water. The two precursors were selected, an appropriate amount of acid was added, and the reaction temperature was also adjusted for each reaction (Table S3, Supporting Information). Dehydration under supercritical conditions promoted the formation of conjugated *sp* ^2^ hybridized domains. Subsequent hydrothermal treatment for 6 h followed by precipitation and separation produced seven solid samples that were redispersed in DI water for characterization. The aqueous solutions have different colors in daylight, and exhibit tunable PL emission from blue to NIR under a 365 nm UV light (**Figure** **2**a). Their PL emission maxima are at 413, 445, 472, 505, 567, 592, and 635 nm, and cover the whole visible spectrum (Figure 2b). Typical blue, green, and red fluorescing CDs samples, called B‐CDs, G‐CDs, and R‐CDs, respectively, were characterized further using an integrating sphere at their optimal excitation wavelengths and the samples show absolute QYs of 38.97%, 75.41%, and 24.99%, respectively. The UV−vis absorption spectra for these samples (Figure 2d–f) show similar absorption in the UV region (200−350 nm), and different absorption at longer wavelengths. Each curve's UV region contains single peaks at 229 and 282 nm, which are corresponding to aromatic *π*–π* transitions in C=C and C=N bonds.^[^ ^24^ ^]^ At longer wavelengths, the three spectra contain distinct absorption bands at 500–700 nm. In contrast to previous CDs in many literatures, the PL emission peaks haven't shifted as the excitation wavelength changes.

Time‐resolved PL decay curves were measured for the different emissions of these samples under 330 nm excitation (Figure 2c). The plots of fluorescence intensity decay reveal the kinetics of the deactivation of electrons from an excited state to the ground state via radiative and nonradiative pathways. The curves observed here can be fitted to two exponentials, similar to those in previous reports. (Table S4 Supporting Information) summarizes the lifetimes, overall contributions, and complete fit parameters. The decays all include a fast component (*τ*_1_), attributed to radiative recombination of the intrinsic states, and two slower components (*τ*_2_) due to surface functional groups. The results for B‐CDs to R‐CDs show that as the average lifetime decreases from 7.20 to 1.87 ns, the percentage of *τ*_1_ increases from 21.87% to 90.97%, suggesting that the core states in the radiative lifetime of these CDs play an increasing role as the PL red‐shift increases.^[^ ^25^, ^26^ ^]^ These findings show that our CDs exhibit excellent uniform optical properties and that their tunable PL emission is linked to their carbon cores, which can be tuned by the reaction temperature and pH.^[^ ^27^ ^]^

A NIR femtosecond pulsed laser (800 nm) was used to study the unusual two‐photon fluorescence properties of B‐CDs, G‐CDs, and R‐CDs. Figure 2g–i show representative two‐photon luminescence spectra of the CDs. The spectra appear similar to the results for single‐photon fluorescence. Comparing various excitation laser powers demonstrated that two‐photon luminescence occurs under pulsed infrared laser excitation. The quadratic relationship with a slope of 1.91 between excitation power and luminescence intensity (Figure S1, Supporting Information) confirms that excitation with two NIR photons produces the multi‐color luminescence of the CDs. The CDs’ luminescence mechanism and carrier relaxation dynamics were explored further by femtosecond transient absorption measurements under excitation at 360 nm. The spectra of CDs probed at 450–720 nm show clear transient absorption at various time delays with a negative and a positive absorption band (Figure S2, Supporting Information). The negative band corresponds to the absorption promoting the CDs’ ground electronic state to the excited electronic state. The positive band corresponds to the absorption of the charge separation state formed by the excited state after light excitation.^[^ ^28^, ^29^ ^]^

**Figure 3:**
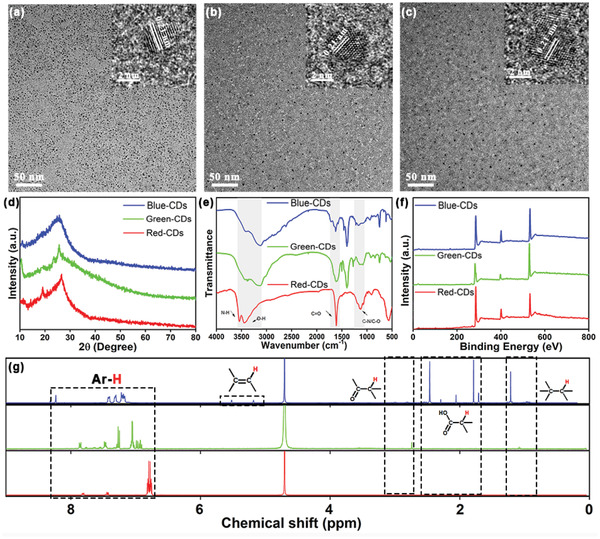
TEM and HRTEM images (inset) of a) B‐CDs, b) G‐CDs, and c) R‐CDs; d) XRD pattern; e) FTIR spectra; f) XPS spectra and g) ^1^H NMR spectrum of the three selected CDs.

Transmission electron microscopy (TEM) images (**Figure** **3**a–c) show well‐dispersed, homogeneous B‐CDs, G‐CDs, and R‐CDs particles with average sizes of 2.78, 3.11, and 4.05 nm, respectively (Figure S3, Supporting Information). High‐resolution TEM (HRTEM) images show well‐resolved fringes 0.21 nm apart due to the (100) planes of graphitic carbon, indicating that the particles had highly crystalline carbon structures.^[^ ^30^ ^]^ Atomic force microscopy image of Figure S4 (Supporting Information) showed that the CDs were monodisperse, with each particle being 1.0–2.0 nm thick, equivalent to three or four graphene layers.^[^ ^31^ ^]^ The increase in the intensity of the broad peak at around 25° in the X‐ray diffraction (XRD) patterns indicates that the degree of graphitization increases from B‐CDs to R‐CDs (Figure 3d). The band‐gap energies were calculated by *E*_g_^opt^ = 1240/*λ*_edge_, where *λ*_edge_ is the onset of the first excitonic absorption band in the direction of longer wavelengths.^[^ ^32^ ^]^ The calculated values decrease gradually from 2.88 to 1.97 eV as CDs size increases, confirming the particle size dependence of the band‐gap energy. The calculated band‐gaps agree well with the experimental results, as shown in Table S5 (Supporting Information).

Fourier transform infrared (FTIR) spectra suggest the presence of many hydrophilic groups (e.g., C=O, C—O, COOH, O—H, and N—H) on the CDs surfaces, making the CDs water soluble (Figure 3e). The C=C, C=O, and C—N stretches indicate that the CDs have polyaromatic structures.^[^ ^33^ ^]^ The FTIR spectra of the CDs show a decrease in the in‐plane O−H vibration at approximately 1380 cm^−1^ from B‐CDs to R‐CDs. In general, the carbonyl and carboxyl groups in the CDs affect the fluorescence.

The full X‐ray photoelectron spectroscopy (XPS) spectra of Figure 3f contain typical peaks at 285, 401, and 532 eV corresponding to C 1s, N 1s, and O 1s, respectively, on the CDs surfaces. The chemical structures and connectivity of the CDs were characterized by ^1^H nuclear magnetic resonance (^1^H NMR) measurements in D_2_O (Figure 3g). Despite the differences in their synthesis, the ^1^H NMR spectra indicate similar conjugate structures with the same type of core structure. The peak at 7–8 ppm is attributed to the benzene ring of oPD, and the conjugated structure appears during the reaction.^[^ ^34^ ^]^ Peaks at lower shifts appear for B‐CDs and G‐CDs attributed to CA attached to oPD, possibly due to the low degree of oxidation and carbonization, which facilitates non‐conjugated *sp* ^2^ and *sp* ^3^ hybridization. The degree of graphitization in R‐CDs is greatest, whereas the other CDs have non‐conjugated structures, which may be caused by the short chains in the CDs. Therefore, a decrease in *sp* ^3^ hybridization red‐shifts the PL emission of the CDs.

The theoretical and experimental results indicate the feasibility of designing multi‐color‐emissive CDs by adjusting the ratio of *sp* ^2^ and *sp* ^3^ hybridized domains. The easiest method is to choose a *sp* ^2^ and a *sp* ^3^ precursor. The *sp* ^2^ hybridization can provide yellow and red emissions, whereas the *sp* ^3^ hybridization provides blue–green emissions. Combining and manipulating the proportions of the hybridized domains via the reaction conditions should produce multi‐color‐emissive CDs.

**Figure 4:**
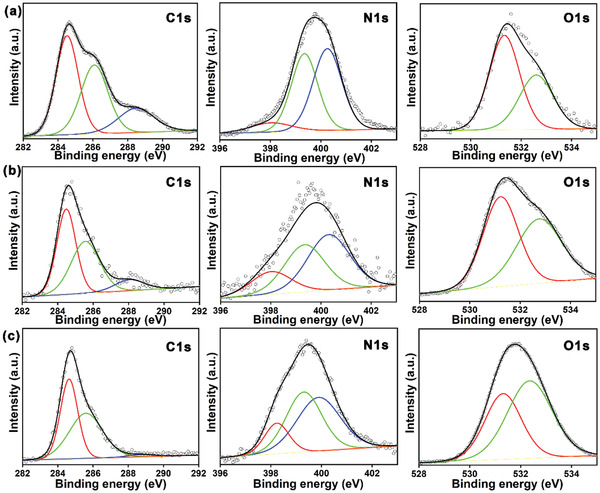
High‐resolution XPS C 1s, N 1s, and O 1s spectra of a) B‐CDs, b) G‐CDs, and c) R‐CDs.

The current work uses CA and oPD precursors. Hydrothermally treating each precursor individually results in that QYs from our CDs are lower than those mentioned in this work, indicating that the combination of the two molecular structures can enhance the QYs. This may be due to CA connecting multiple oPD molecules through the carboxyl groups. Previously reported CA‐derived CDs have shown blue emission, whereas oPD‐derived CDs have shown red emission. Changing the reaction conditions can tailor the CDs’ emission between blue–green and yellow–red by increasing the dominance of oPD, i.e., the *sp* ^2^ hybrid structure can red‐shift the emission wavelength. Surface analysis by high‐resolution XPS was further used to investigate the factors affecting the luminescence of the CDs, as shown in **Figure** **4**. The results show that the samples contain the same elements. The C 1s band has three component peaks corresponding to *sp* ^2^ carbon (C=C, 284.6 eV), *sp* ^3^ carbon (C−O/C−N, 286.1 eV), and carbonyl groups (C=O, 288.4 eV). The N 1s band also has three component peaks, at 399.0, 400.2, and 401.1 eV, corresponding to pyridinic N, pyrrolic N, and graphitic N, respectively. The two‐component O 1s peaks at 531.5 and 532.5 eV arose from C=O and C−O. The *sp* ^2^ carbon content increases gradually from 44.4% in B‐CDs to 52.91% in R‐CDs (Table S6, Supporting Information). The content of graphitic N increases more steeply, also indicating that the growth of *sp* ^2^ hybridized domains in the CDs follows the dehydration reaction. This result agrees with the increases in particle size and graphitization. The O 1s spectra show that the amount of C=O bonds in the CDs, corresponding to the degree of oxidation, increases with their red‐shifting emission. Overall, the CDs have extensive conjugated *sp* ^2^ domains including many surface groups containing oxygen and nitrogen. The particle size and extent of *sp* ^2^ hybridization increase as the PL emission is red‐shifted.

These findings demonstrate that the CDs comprise carbon cores with *π*‐conjugated domains surrounded by amorphous surface regions and that the particles’ degree of oxidation increases as their PL red‐shifts. The effects of size and *sp* ^2^/*sp* ^3^ hybridization ratio are also important. Even though the PL mechanisms of CDs are not fully understood, their strong PL is a powerful motivation for continuing research. There are two main models proposed for CDs: the first considers band‐gap transitions in conjugated *π*‐domains, and the second is based on surface defects. Several studies have used the first model to attribute the red‐shifting of CDs PL to quantum size effects. Here, the *sp* ^2^/*sp* ^3^ hybridized domains and particle size were considered the main factors that controlled the PL (Table S7, Supporting Information).

To illustrate the contributions of the different functional groups to the PL of CDs, their surface structures were controlled by using NaBH_4_, which is commonly used to reduce aldehydes and ketones to alcohols, and by using NaOH, which reacts with acidic groups on the CDs surfaces.^[^ ^35^ ^]^ Adding NaBH_4_ and NaOH to the CDs affects the fluorescence intensity, but not the emission peak position, indicating that the surface state is inert (Figures S5 and S6, Supporting Information). Therefore, the emission of our CDs should be controlled by their internal structure and size.

**Figure 5:**
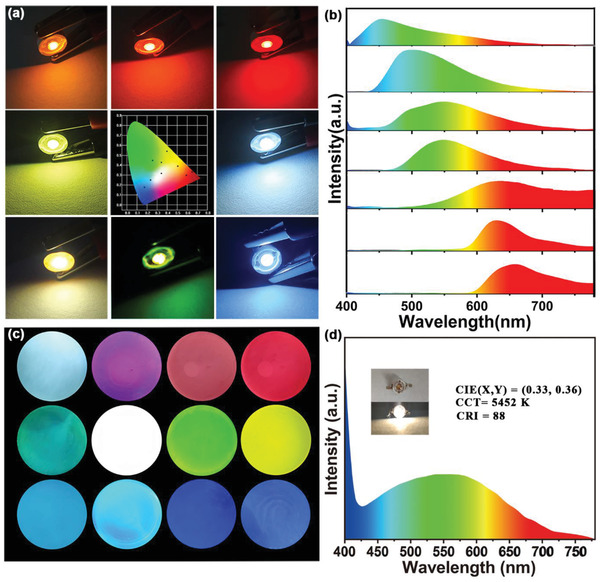
a) The optical photograph of multi‐color LEDs and the CIE color coordinates; b) The corresponding emission spectra of the multi‐color LEDs; c) PL photographs of selected CDs, and their mixtures in epoxy composite films under 365 nm UV irradiation; d) The emitting light spectrum of white LEDs (inset: optical photograph of the white LEDs).

Given the CDs’ interesting multi‐colored solution emission, they were used to make multi‐color emissive films. Thermosetting epoxy resin is commonly used in adhesives, paints, and electrical sealants, for example, for encapsulating LEDs chips.^[^ ^36^, ^37^ ^]^ The CDs were initially dissolved in an epoxy hardener. The resulting clear solution was mixed with hardener at a 2.5:1 volume ratio. Curing the mixture for 4 h at room temperature formed a CDs/epoxy composite, which was used to encapsulate LEDs chips. The photographs in **Figure** **5**a show various monochromatic down‐conversion LEDs made by coating 365 nm chips with the composite. The points on the CIE color plot and the spectra in Figure 5b show that these LEDs cover the entire visible spectrum. Among them, The CRI of red LEDs is 82.49, which is a relatively high level among the current photoluminescence LEDs based on CDs, even better than many white LEDs. In addition, the CIE of the red LEDs is (0.62, 0.34), which is closer to the standard red than most red LEDs based on CDs (Table S8 Supporting Information). We further conducted a stability test, and after 6 months of storage, the performance of the red LEDs remained stable (Figure S7a, Supporting Information). To test the stability of the red LEDs device during operation, the device is kept continuously illustrating over 7 d (4 V, 20 mA). The emitting light spectra of the red LEDs at the beginning of and after 7 d are shown in the (Figure S7b Supporting Information). The emitting light spectrum has insignificant change compared with most organic dyes. This indicates that the CDs based LEDs has excellent stability. The optical micrographs in Figure 5c are of composite discs. The discs showed a range of colors because of the added CDs. All the discs were transparent and emitted various uniform colors from blue to red under the corresponding excitation light (Figure S8, Supporting Information). A white LED was made by encapsulating a UV chip (365 nm) in an epoxy composite containing a mixture of the B‐CDs, G‐CDs, and R‐CDs. Adjusting the proportions of the three CDs can tune the LEDs' emission to pure white with CIE color coordinates of (0.33, 0.36) (Figure 5d). The resulting correlated color temperature and color rendering index (CRI) were 5452 K and 88, which suggests that our method is suitable for fabricating white LEDs with high CRI.

## 3 Conclusion

A size‐dependent model was developed that comprised CDs with *sp* ^3^‐hybridized carbon cores containing small areas of *sp* ^2^‐hybridized carbon. The origin of PL in the CDs was investigated via first‐principles DFT calculation. The results showed that controlling the distributions of *sp* ^2^ and *sp* ^3^ hybridization red‐shifted the CDs emission. The calculated results and theory were used to design a method for making full‐spectrum‐emitting CDs with two‐photon fluorescence. The experiments showed the predictions to be appropriate, and demonstrated the feasibility of the method, which could be used in various fields. Finally, the full‐spectrum‐emitting CDs were used to make multi‐color LEDs and films. Our simple analytical model replicated the major optical properties of the CDs and represents a universal method for guiding further experiments on full‐spectrum‐emitting CDs.

## 4 Experimental Section

##### Materials

*o*‐phenylenediamine (oPD), Citric Acid (CA), NaOH, HCl were purchased from Sigma‐Aldrich Co. LLC. All chemicals were used directly without further purification. The redistilled water used in this experiment was purified via the SZ‐93A water purification system. All the reagents were analytical grade and utilized without further purification.

##### Preparation of Multi‐Color‐Emissive CDs

Multi‐color‐emissive carbon dots (CDs) were synthesized by using CA and oPD by hydrothermal at different temperatures and pH. Violet‐CDs: oPD (108 mg), CA (192 mg), 10 mL of redistilled water (pH = 6) were added to a 25 mL autoclave and heated at 240 °C for 6 h. Blue‐CDs: oPD (108 mg), CA (192 mg), 10 mL of redistilled water (pH = 2) were added to a 25 mL autoclave and heated at 240 °C for 6 h. Cyan‐CDs: oPD (108 mg), CA (192 mg), 10 mL of redistilled water (pH = 1) were added to a 25 mL autoclave and heated at 140 °C for 6 h. Green‐CDs: oPD (108 mg), CA (192 mg), 10 mL of redistilled water (pH = 2) were added to a 25 mL autoclave and heated at 180 °C for 6 h. Yellow‐CDs: oPD (108 mg), CA (192 mg), 10 mL of redistilled water (pH = 6) were added to a 25 mL autoclave and heated at 140 °C for 6 h. Orange‐CDs: oPD (108 mg), CA (192 mg), 10 mL of redistilled water (pH = 6) were added to a 25 mL autoclave and heated at 120 °C for 6 h. Red‐CDs: oPD (108 mg), 1 mL H_2_SO_4_ and 10 mL of redistilled water were added to a 25 mL autoclave and heated at 200 °C for 6 h. After cooling to room temperature, the solution was centrifuged at 10 000 rpm for 5 min, then filtrated through a 0.22 µm filter membrane and finally dialyzed with membrane (1000 Da) for 24 h to remove the salt and acid.

##### Preparation of CDs‐NaBH_4_ and CDs‐NaOH

Add 100 µL of CDs solution (2 mg mL^−1^) to 2 mL NaBH_4_ or NaOH solution with different concentrations. The spectrum was tested using a fluorescence spectrophotometer.

For more experimental details, please refer to the Supporting Information.

## Conflict of Interest

The authors declare no conflict of interest.

1

N.
Mishra
, 
N. J.
Orfield
, 
F.
Wang
, 
Z.
Hu
, 
S.
Krishnamurthy
, 
A. V.
Malko
, 
J. L.
Casson
, 
H.
Htoon
, 
M.
Sykora
, 
J. A.
Hollingsworth
, Nat. Commun.
2017, 8, 15083.2849777610.1038/ncomms15083PMC54372952

B.
Huang
, 
W. C.
Chen
, 
Z.
Li
, 
J.
Zhang
, 
W.
Zhao
, 
Y.
Feng
, 
B. Z.
Tang
, 
C. S.
Lee
, Angew. Chem., Int. Ed.
2018, 57, 12473.10.1002/anie.201806800300399083

K.‐K.
Liu
, 
R.
Zhou
, 
Y.‐C.
Liang
, 
C.‐Z.
Guo
, 
Z.‐K.
Xu
, 
C.‐X.
Shan
, 
L.
Li
, 
D.‐Z.
Shen
, Sci. China Mater.
2018, 61, 1191.4

X.
Li
, 
J.
Cui
, 
Q.
Ba
, 
Z.
Zhang
, 
S.
Chen
, 
G.
Yin
, 
Y.
Wang
, 
B.
Li
, 
G.
Xiang
, 
K. S.
Kim
, 
H.
Xu
, 
Z.
Zhang
, 
H. L.
Wang
, Adv. Mater.
2019, 31, 1900613.10.1002/adma.201900613309937855

W.
Zhang
, 
J.
Yao
, 
Y. S.
Zhao
, Acc. Chem. Res.
2016, 49, 1691.2756039010.1021/acs.accounts.6b002096

W.
Zhou
, 
D.
Li
, 
C.
Xiong
, 
R.
Yuan
, 
Y.
Xiang
, ACS Appl. Mater. Interfaces
2016, 8, 13303.2719574710.1021/acsami.6b031657

Y.
Zhuang
, 
L.
Wang
, 
Y.
Lv
, 
T. L.
Zhou
, 
R. J.
Xie
, Adv. Funct. Mater.
2018, 28, 1705769.8

S.
Liu
, 
H.
Ming
, 
J.
Cui
, 
S.
Liu
, 
W.
You
, 
X.
Ye
, 
Y.
Yang
, 
H.
Nie
, 
R.
Wang
, J. Phys. Chem. C
2018, 122, 16289.9

X.
Zhuang
, 
Y.
Ouyang
, 
X.
Wang
, 
A.
Pan
, Adv. Opt. Mater.
2019, 7, 1900071.10

G.
Pan
, 
X.
Bai
, 
D.
Yang
, 
X.
Chen
, 
P.
Jing
, 
S.
Qu
, 
L.
Zhang
, 
D.
Zhou
, 
J.
Zhu
, 
W.
Xu
, Nano Lett.
2017, 17, 8005.2918287710.1021/acs.nanolett.7b0457511

C.
Li
, 
J.
Zhang
, 
S.
Zhang
, 
Y.
Zhao
, Angew. Chem., Int. Ed.
2019, 58, 1643.10.1002/anie.2018121463041870012

M.
Yu
, 
P.
Zhang
, 
B. P.
Krishnan
, 
H.
Wang
, 
Y.
Gao
, 
S.
Chen
, 
R.
Zeng
, 
J.
Cui
, 
J.
Chen
, Adv. Funct. Mater.
2018, 28, 1804759.13

C.
Xia
, 
S.
Zhu
, 
T.
Feng
, 
M.
Yang
, 
B.
Yang
, Adv. Sci.
2019, 6, 1901316.10.1002/advs.201901316PMC68919143183231314

W.
Li
, 
Y.
Liu
, 
B.
Wang
, 
H.
Song
, 
Z.
Liu
, 
S.
Lu
, 
B.
Yang
, Chin. Chem. Lett.
2019, 30, 2323.15

W.
Meng
, 
X.
Bai
, 
B.
Wang
, 
Z.
Liu
, 
S.
Lu
, 
B.
Yang
, Energy Environ. Mater.
2019, 2, 172.16

K.
Jiang
, 
S.
Sun
, 
L.
Zhang
, 
Y.
Lu
, 
A.
Wu
, 
C.
Cai
, 
H.
Lin
, Angew. Chem., Int. Ed.
2015, 54, 5360.10.1002/anie.2015011932583229217

H.
Ding
, 
S.‐B.
Yu
, 
J.‐S.
Wei
, 
H.‐M.
Xiong
, ACS Nano
2016, 10, 484.2664658410.1021/acsnano.5b0540618

X.
Miao
, 
D.
Qu
, 
D.
Yang
, 
B.
Nie
, 
Y.
Zhao
, 
H.
Fan
, 
Z.
Sun
, Adv. Mater.
2018, 30, 1704740.10.1002/adma.2017047402917838819

F.
Yuan
, 
Z.
Wang
, 
X.
Li
, 
Y.
Li
, 
Z.
Tan
, 
L.
Fan
, 
S.
Yang
, Adv. Mater.
2017, 29, 1604436.10.1002/adma.2016044362787901320

S.
Lu
, 
L.
Sui
, 
M.
Wu
, 
S.
Zhu
, 
X.
Yong
, 
B.
Yang
, Adv. Sci.
2019, 6, 1801192.10.1002/advs.201801192PMC63430633069318021

H.
Ding
, 
J.‐S.
Wei
, 
P.
Zhang
, 
Z.‐Y.
Zhou
, 
Q.‐Y.
Gao
, 
H.‐M.
Xiong
, Small
2018, 14, 1800612.10.1002/smll.2018006122970910422

N. V.
Tepliakov
, 
E. V.
Kundelev
, 
P. D.
Khavlyuk
, 
Y.
Xiong
, 
M. Y.
Leonov
, 
W.
Zhu
, 
A. V.
Baranov
, 
A. V.
Fedorov
, 
A. L.
Rogach
, 
I. D.
Rukhlenko
, ACS Nano
2019, 13, 10737.3141186010.1021/acsnano.9b0544423

M. A.
Sk
, 
A.
Ananthanarayanan
, 
L.
Huang
, 
K. H.
Lim
, 
P.
Chen
, J. Mater. Chem. C
2014, 2, 6954.24

S.
Lu
, 
L.
Sui
, 
J.
Liu
, 
S.
Zhu
, 
A.
Chen
, 
M.
Jin
, 
B.
Yang
, Adv. Mater.
2017, 29, 1603443.10.1002/adma.2016034432819536925

F.
Ehrat
, 
S.
Bhattacharyya
, 
J.
Schneider
, 
A.
Lof
, 
R.
Wyrwich
, 
A. L.
Rogach
, 
J. K.
Stolarczyk
, 
A. S.
Urban
, 
J.
Feldmann
, Nano Lett.
2017, 17, 7710.2918871110.1021/acs.nanolett.7b0386326

Y.
Zhang
, 
R.
Yuan
, 
M.
He
, 
G.
Hu
, 
J.
Jiang
, 
T.
Xu
, 
L.
Zhou
, 
W.
Chen
, 
W.
Xiang
, 
X.
Liang
, Nanoscale
2017, 9, 17849.2911627410.1039/c7nr05363k27

L.
Wang
, 
Y.
Wang
, 
T.
Xu
, 
H.
Liao
, 
C.
Yao
, 
Y.
Liu
, 
Z.
Li
, 
Z.
Chen
, 
D.
Pan
, 
L.
Sun
, Nat. Commun.
2014, 5, 5357.2534834810.1038/ncomms635728

S.
Lu
, 
G.
Xiao
, 
L.
Sui
, 
T.
Feng
, 
X.
Yong
, 
S.
Zhu
, 
B.
Li
, 
Z.
Liu
, 
B.
Zou
, 
M.
Jin
, 
J. S.
Tse
, 
H.
Yan
, 
B.
Yang
, Angew. Chem., Int. Ed.
2017, 56, 6187.10.1002/anie.2017007572837852029

Q.
Wang
, 
S.
Zhang
, 
B.
Wang
, 
X.
Yang
, 
B.
Zou
, 
B.
Yang
, 
S.
Lu
, Nanoscale Horiz.
2019, 4, 1227.30

W.
Li
, 
Y.
Liu
, 
M.
Wu
, 
X.
Feng
, 
S. A. T.
Redfern
, 
Y.
Shang
, 
X.
Yong
, 
T.
Feng
, 
K.
Wu
, 
Z.
Liu
, 
B.
Li
, 
Z.
Chen
, 
J. S.
Tse
, 
S.
Lu
, 
B.
Yang
, Adv. Mater.
2018, 30, 1800676.10.1002/adma.2018006762992079531

S.
Zhu
, 
Q.
Meng
, 
L.
Wang
, 
J.
Zhang
, 
Y.
Song
, 
H.
Jin
, 
K.
Zhang
, 
H.
Sun
, 
H.
Wang
, 
B.
Yang
, Angew. Chem., Int. Ed.
2013, 52, 3953.10.1002/anie.2013005192345067932

F.
Yuan
, 
T.
Yuan
, 
L.
Sui
, 
Z.
Wang
, 
Z.
Xi
, 
Y.
Li
, 
X.
Li
, 
L.
Fan
, 
Z. a.
Tan
, 
A.
Chen
, Nat. Commun.
2018, 9, 2249.2988487310.1038/s41467-018-04635-5PMC599380033

L.
Vallan
, 
E. P.
Urriolabeitia
, 
F.
Ruiperez
, 
J. M.
Matxain
, 
R.
Canton‐Vitoria
, 
N.
Tagmatarchis
, 
A. M.
Benito
, 
W. K.
Maser
, J. Am. Chem. Soc.
2018, 140, 12862.3021154710.1021/jacs.8b0605134

B.
Wang
, 
J.
Li
, 
Z.
Tang
, 
B.
Yang
, 
S.
Lu
, Sci. Bull.
2019, 64, 1285.10.1016/j.scib.2019.07.0213665961035

C.
Liu
, 
L.
Bao
, 
M.
Yang
, 
S.
Zhang
, 
M.
Zhou
, 
B.
Tang
, 
B.
Wang
, 
Y.
Liu
, 
Z.‐L.
Zhang
, 
B.
Zhang
, J. Phys. Chem. Lett.
2019, 10, 3621.3119916210.1021/acs.jpclett.9b0133936

D.
Qu
, 
M.
Zheng
, 
J.
Li
, 
Z.
Xie
, 
Z.
Sun
, Light: Sci. Appl.
2015, 4, 364.37

H.
Song
, 
X.
Liu
, 
B.
Wang
, 
Z.
Tang
, 
S.
Lu
, Sci. Bull.
2019, 64, 1788.10.1016/j.scib.2019.10.00636659538

## Supporting information

Supporting Information
