# Supplementary material for: Rational Design of Multi‐Color‐Emissive Carbon Dots in a Single Reaction System by Hydrothermal
Source: Adv Sci (Weinh). 2020 Nov 23;8(1):2001453. doi: 10.1002/advs.202001453 (PMC7788586; doi:10.1002/advs.202001453)
Supplement: Supplementary file 1 — Supporting Information [file ADVS-8-2001453-s001.pdf]

© 2020 Wiley-VCH GmbH

ADVANCED SCIENCE Open Access

Supporting Information

for *Adv. Sci.*, DOI: 10.1002/advs.202001453

Rational Design of Multi-color-emissive Carbon Dots in a Single Reaction System by Hydrothermal

*Boyang Wang, Jingkun Yu, Laizhi Sui, Shoujun Zhu, Zhiyong Tang, Bai Yang, and Siyu Lu**

Supporting Information

Rational Design of Multi-color-emissive Carbon Dots in a Single Reaction System by Hydrothermal

*Boyang Wang, Jingkun Yu, Laizhi Sui, Shoujun Zhu, Zhiyong Tang, Bai Yang, and Siyu Lu**

Experimental Procedures

Characterization of CDs: Transmission electron microscopy (TEM) images were acquired with a FEI TECNAIG2F20-S-TWIN electron microscope. X-ray photoelectron spectroscopy (XPS) measurements used a Thermo Fisher ESCALAB 250Xi surface analysis system. X-ray diffraction (XRD) patterns were obtained using an X-ray diffractometer (PANalytical, X'Pert PRO). The absorption and fluorescence spectra of the CDs were recorded on a Persee TU-1810PC spectrophotometer and Shimadzu RF-6000 fluorescence spectrophotometer at room temperature, respectively. Fourier transform infrared spectroscopy (FTIR) was performed on a Nexus 470 (Thermo Fisher) spectrometer.

Femtosecond transient absorption setup: A regeneratively amplified Ti: sapphire laser system (Coherent Libra, 50fs, 1kHz) provides the fundamental light source. The pump pulse (400 nm) is generated by focusing a portion of fundamental light into BBO crystal. In order to avoid the influence of rotational relaxation effects on dynamics, the polarization of pump pulse is randomized by depolarizing plate. The other fundamental pulse provides broadband probe pulse (white light continuum) that is produced by focusing 800 nm fundamental light into sapphire plate (3mm). The pump and probe beams are overlapped in the sample with crossing areas of 600 μm and 150 μm. After passing through the sample, the probe pulse is focused into optical fiber that is coupled to spectrometer (AvaSpec-1650F). The energy of 400 nm excitation pulse is adjusted to about 1.5 μJ/pulse by a neutral density optical filter. The pump pulse is chopped at 500 Hz to acquire pumped (signal) and un-pumped (reference) probe spectra, and the ΔOD spectrum can be obtained by processing them. The solutions are placed in 2 mm optical path length quartz cuvette. Both the instrument response function (100 fs) and temporal chirp in the probe light are

determined by measuring the cross modulation of ethanol. The group velocity dispersion effect on the experiment data is corrected by home-made chirp program. For each measurement, the pump-probe delay scan is repeated three times to give the averaged experiment data.

Computational details: All the calculations are performed with Gaussian 09 program. The ground-state geometries of carbon dots were optimized by DFT B3LYP (Becke's three-parameter hybrid function with the non-local correlation of Lee-Yang-Parr) functional, 6-31G(d) basis set (B3LYP/6-31G(d). The absorption spectra of carbon dots were calculated using TDDFT method (at the B3LYP/6-31G(d) level) based on optimized ground-state geometries. The first excited state was optimized using TDDFT method to calculate the emission energy (wavelength) which is the energy difference between the ground and the first excited state.

Fabrication and the character of LEDs from CDs: GaN LEDs chips without phosphor coating were purchased from Advanced Optoeletronic Technology CO., Ltd. The emission peak of GaN LEDs chips located at 365 nm with the operating voltage of 4.0 V. The composite of polydimethylsiloxane and CDs is then used directly as a phosphor to drip onto the LED chip. The LED chip was then placed in a 60 °C oven for 2 hours. The emission spectra of LEDs were measured by combining a Spectra scan PR-650 spectrophotometer with an integrating sphere and a computer-controlled direct current power supply Keithley model 2400 voltage current source under ambient condition at room temperature. The color of the light was identified by the CIE (Commission Internationale de L'Eclairage1931) calorimeter system. All measurements were performed under dark condition.

Results and Discussion

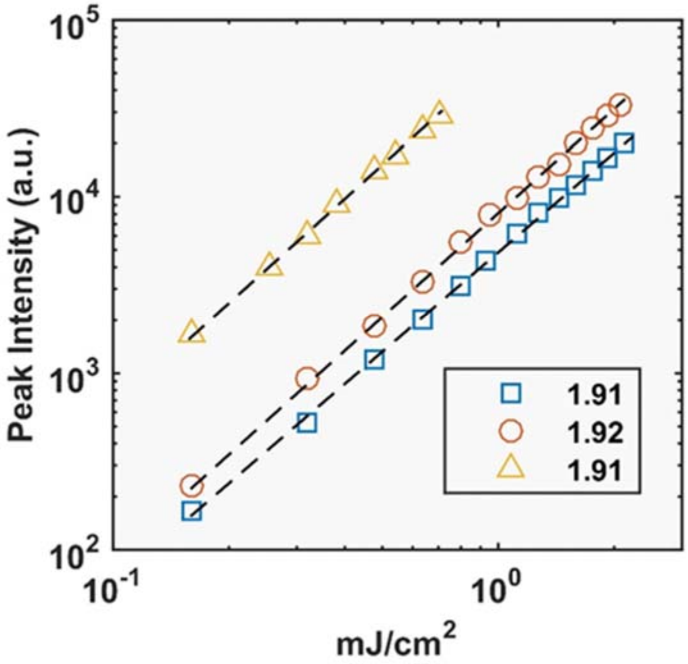

Figure S1. Relationship between the two-photon emission intensity and the square of laser power.

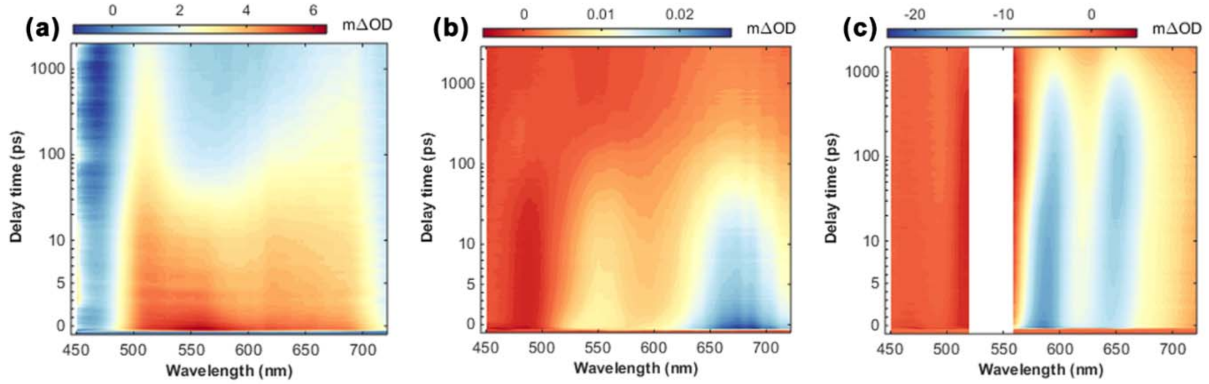

Figure S2. Two-dimensional pseudo-colour map of the TA spectra of the B-CDs(a), G-CDs(b), R-CDs(c).

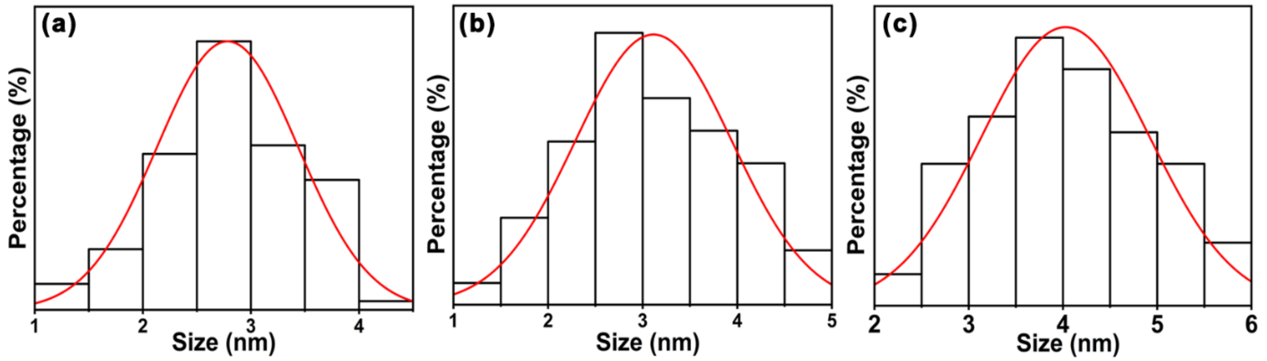

Figure S3. Particle size distribution diagrams of the B-CDs(a), G-CDs(b), R-CDs(c).

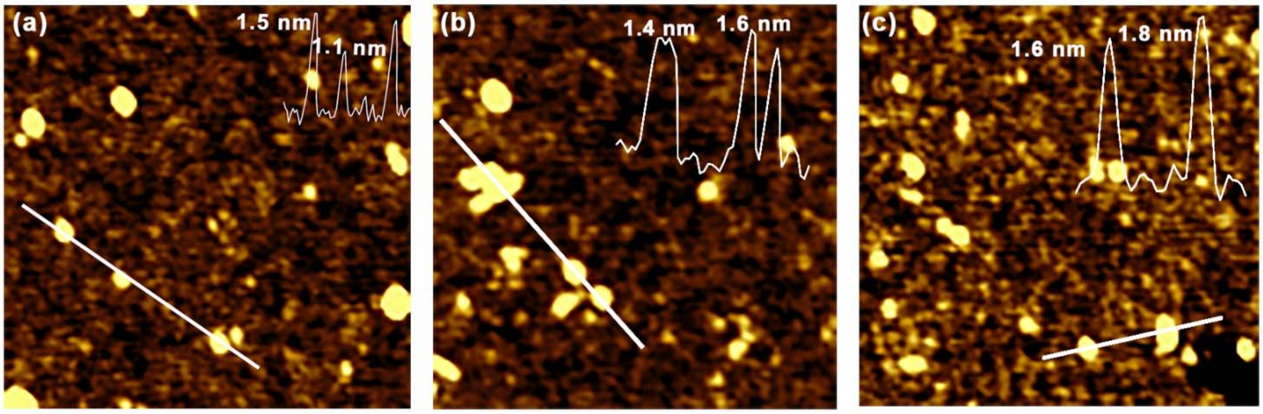

Figure S4. AFM images of the B-CDs(a), G-CDs(b), R-CDs(c).

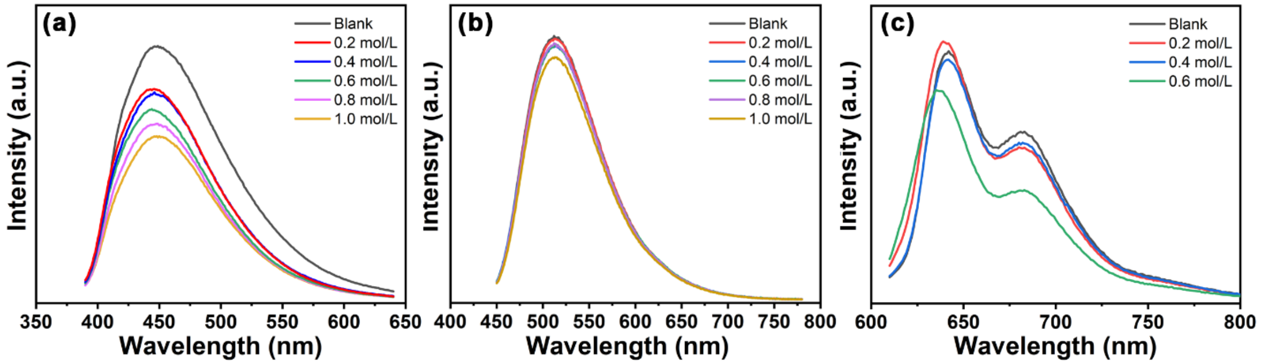

Figure S5. PL spectra of the B-CDs(a), G-CDs(b), R-CDs(c) in different concentrations of $NaBH_4$.

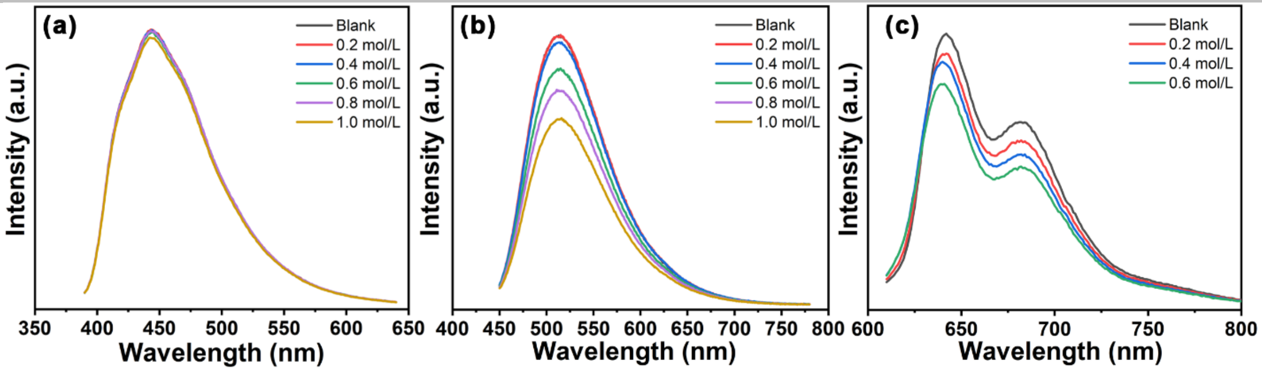

Figure S6. PL spectra of the B-CDs(a), G-CDs(b), R-CDs(c) in different concentrations of NaOH.

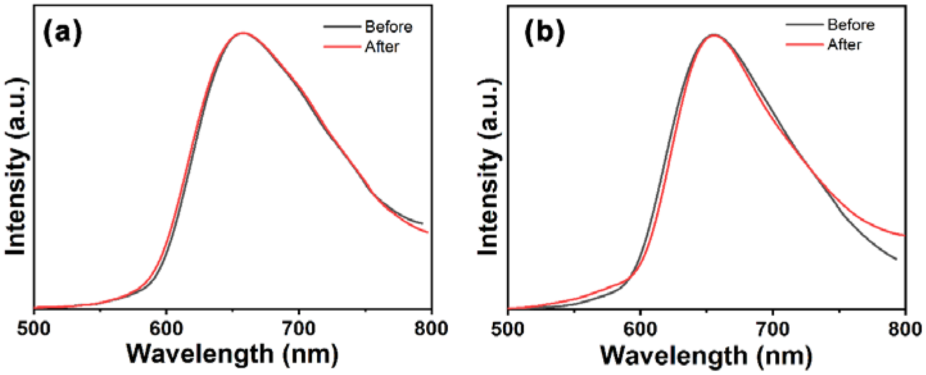

Figure S7. (a) The emitting spectra of the red LED at initial and after storage over 6 months, (b) The emitting spectra of the white LED at initial and after illustrating over 7 d.

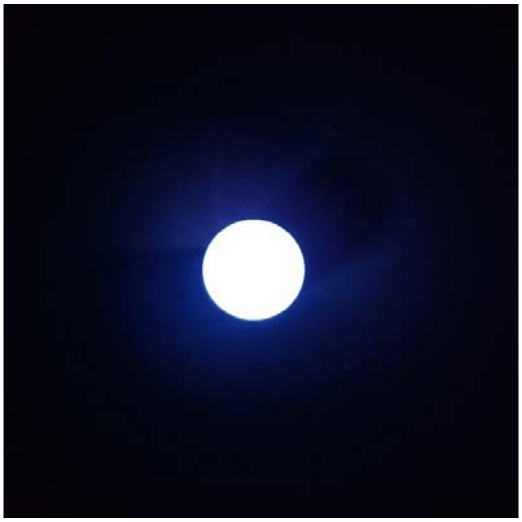

Figure S8. Photograph of the white color discs with brilliantly emission.

Table S1. HOMO and LUMO states of the established model by increasing the aromatic rings

Number of aromatic rings (N)	Bandgap (eV)	Excitation energy (nm)]
0	5.64	219.88
7	2.97	417.12
10	2.56	484.69
19	2.18	567.97
22	2.02	614.09
24	1.97	626.87

Table S2. HOMO and LUMO states of the established model by increasing the ratio of sp^2/sp^3 hybridized domains

Ratio of sp^2/sp^3 hybridized domains	Bandgap (eV)	Excitation energy (nm)]
0.13	3.29	376.45
0.16	2.84	435.27
0.20	2.55	485.86
0..23	2.14	577.72
0.26	1.96	632.83

Table S3. The color of PL emission of CDs at different pH values and different reaction temperatures.

	120 ℃	140 ℃	180 ℃	240 ℃
pH=1	Yellow-Orange	Cyan	Blue-Green	Blue
pH=2	Yellow-Orange	Cyan	Green	Blue
pH=5	Orange	Yellow-Orange	Blue	Violet
pH=6	Orange	Yellow	Blue-Violet	Violet

Table S4. Summary of PL lifetimes of the three selected products as indicated.

Sample	λem (nm)	τ_1 (ns)	[B] (%)	τ_2 (ns)	[B] (%)	τ(avg)	χ^2
B-CDs	446	1.07	21.87	8.91	78.13	7.20	1.15
G-CDs	505	2.46	45.30	10.44	54.70	6.83	1.14
R-CDs	635	1.41	90.97	6.48	9.03	1.87	1.24

Table S5. Estimation of the energy levels of the three selected CDs

Sample	λedge (nm)	E_g^{opt} (eV)	Calculated band-gaps (eV)
B-CDs	430	2.88	2.84
G-CDs	491	2.52	2.55
R-CDs	629	1.97	1.96

Table S6. XPS data analyses of the C1s spectra of the three selected CDs

Sample	C=C (%)	C−O/C−N (%)	C=O (%)
B-CPs	44.4	37.6	18.14
G-CDs	47.38	43.15	9.48
R-CDs	52.91	44.79	2.3

Table S7. Some literature concerning CDs with tunable photoluminescence.

Precursor	Synthetic Method	PL Peak (nm)	QY (%)	PL mechanism	References
Carbon fibres	Acid oxidation	430-610	2-21	Size effects<br>Degree of surface oxidation	[1]
CA<br>PEI-EC	Hydrothermal	400-710	5-20	Surface groups	[2]
o-,p-,m-phenylenediamine	Solvothermal	423-604	10-26	Size effects<br>Nitrogen content	[3]
Urea<br>p-phenylenediamine	Hydrothermal	440-625	9-23	Degree of surface oxidation	[4]
Citric acid<br>Urea	Solvothermal	460-630	4-13	Graphitic N content	[5]
Citric acid<br>Urea	Solvothermal	448-638	8-32	Size effects	[6]
Citric acid<br>Diaminonaphthalene	Solvothermal	430-604	12-75	Size effects	[7]
L-glutamic<br>o-phenylenediamine	Solvothermal	443-745	13-54	Size effects<br>Graphitic N content	[8]
Citric acid<br>Urea	Solvothermal	430-630	13-52	Graphitization<br>Surface sate	[9]
Tobias acid<br>o-phenylenediamine	Solvothermal	400-600	50-65	Degree of surface oxidation	[10]
Citric acid<br>o-phenylenediamine	Hydrothermal	413-635	25-75	Size effects<br>sp^2/sp^3 hybridization	Our work

Table S8. Device Performances of previously reported white and red color CDs/Based Phosphor LEDs.

Color	CIE (x, y)	CCT (K)	CRI	Stability	References
White	(0.367, 0.314)	3867	70.6	-	[11]

White	(0.29, 0.30)	8526	83	-	[12]
White	(0.34,0.31)	5048	82.4	-	[6]
White	(0.33, 0.33)	5746	83	-	[13]
White	(0.397, 0.428)	3949	70	-	[14]
White	(0.38, 0.49)	4492	76.6	-	[15]
White	(0.33, 0.34)	5590	83.2	-	[16]
White	(0.338, 0.335)	5231	76	-	[17]
Red	(0.62, 0.34)	1221	82.5	7 d	Our work
Red	(0.62, 0.36)	-	-	-	[6]
Red	(0.61, 0.34)	-	-	-	[9]
Red	(0.48, 0.35)	1881	77	-	[12]
Red	(0.59, 0.41)	-	-	-	[18]
Red	(0.60, 0.34)	-	-	-	[19]
Red	(0.496, 0.402)	-	-	-	[20]
Red	(0.63, 0.37)	-	-	-	[21]
Red	(0.51, 0.47)	-	-	-	[22]

References

[1] L. Bao, C. Liu, Z. L. Zhang, D. W. Pang, *Adv. Mater.* **2015**, *27*, 1663-1667.

[2] S. Hu, A. Trinchi, P. Atkin, I. Cole, *Angew. Chem. Int. Ed.* **2015**, *54*, 2970-2974.

[3] K. Jiang, S. Sun, L. Zhang, Y. Lu, A. Wu, C. Cai, H. Lin, *Angew. Chem. Int. Ed.* **2015**, *54*, 5360-5363.

[4] H. Ding, S. B. Yu, J. S. Wei, H. M. Xiong, *ACS Nano* **2016**, *10*, 484-491.

[5] K. Hola, M. Sudolska, S. Kalytchuk, D. Nachtigallova, A. L. Rogach, M. Otyepka, R. Zboril, *ACS Nano* **2017**, *11*, 12402-12410.

[6] Z. Tian, X. Zhang, D. Li, D. Zhou, P. Jing, D. Shen, S. Qu, R. Zboril, A. L. Rogach, *Adv. Optical Mater.* **2017**, *5*, 1700416.

[7] F. Yuan, Z. Wang, X. Li, Y. Li, Z. Tan, L. Fan, S. Yang, *Adv. Mater.* **2017**, *29*, 1604436.

[8] H. Ding, J.-S. Wei, P. Zhang, Z.-Y. Zhou, Q.-Y. Gao, H.-M. Xiong, *Small* **2018**, *14*, 1800612.

[9] X. Miao, D. Qu, D. Yang, B. Nie, Y. Zhao, H. Fan, Z. Sun, *Adv. Mater.* **2018**, *30*, 1704740.

[10] Y. Liu, M. Zhang, Y. Wu, R. Zhang, Y. Cao, X. Xu, X. Chen, L. Cai, Q. Xu, *Chem. Commun.* **2019**, *55*, 12164-12167.

[11] D. Chen, H. Gao, X. Chen, G. Fang, S. Yuan, Y. Yuan, *ACS Photonics* **2017**, *4*, 2352-2358.

[12] T. Feng, Q. Zeng, S. Lu, X. Yan, J. Liu, S. Tao, M. Yang, B. Yang, *ACS Photonics* **2017**, *5*, 502-510.

[13] Z. Han, K. Wang, F. Du, Z. Yin, Z. Xie, S. Zhou, *J. Mater. Chem. C* **2018**, *6*, 9631-9635.

[14] J. Ren, J. Sun, X. Sun, R. Song, Z. Xie, S. Zhou, *Adv. Optical Mater.* **2018**, *6*, 1800115.

[15] C.-L. Shen, J.-H. Zang, Q. Lou, L.-X. Su, Z. Li, Z.-Y. Liu, L. Dong, C.-X. Shan, *Carbon* **2018**, *136*, 359-368.

[16] Y. Zhan, B. Shang, M. Chen, L. Wu, *Small* **2019**, *15*, 1901161.

[17] J. Zhu, X. Bai, X. Chen, H. Shao, Y. Zhai, G. Pan, H. Zhang, E. V. Ushakova, Y. Zhang, H. Song, A. L. Rogach, *Adv. Optical Mater.* **2019**, *7*, 1801599.

[18] J. Shao, S. Zhu, H. Liu, Y. Song, S. Tao, B. Yang, *Adv. Sci.* **2017**, *4*, 1700395.

[19] Z. Bi, T. Li, H. Su, Y. Ni, L. Yan, *ACS Sustain. Chem. Eng.* **2018**, *6*, 9314-9323.

[20] B. E. Kwak, H. J. Yoo, D. H. Kim, *Adv. Optical Mater.* **2019**, *7*, 1900932.

[21] W. Cai, T. Zhang, M. Xu, M. Zhang, Y. Guo, L. Zhang, J. Street, W.-J. Ong, Q. Xu, *J. Mater. Chem. C* **2019**, *7*, 2212-2218.

[22] A. Pyne, S. Layek, A. Patra, N. Sarkar, *J. Mater. Chem. C* **2019**, *7*, 6414-6425.
